# The prevalence, risk factors and prognostic implications of dysphagia in elderly patients undergoing hip fracture surgery in Korea

**DOI:** 10.1186/s12877-019-1382-x

**Published:** 2019-12-18

**Authors:** Seong-Eun Byun, Kyeu Back Kwon, Sang Ho Kim, Seung-Jae Lim

**Affiliations:** 1 Department of Orthopaedic Surgery, CHA Bundang Medical Center, CHA University, Seongnam-si, Gyeonggi-do Republic of Korea; 2 Department of Orthopaedic Surgery, Samsung Medical Center, Sungkyunkwan University School of Medicine, 81 Irwon-ro, Gangnam-gu, Seoul, 06351 Republic of Korea

**Keywords:** Dysphagia, Hip fracture, Frailty, Prevalence, Prognosis

## Abstract

**Background:**

Dysphagia is prevalent in geriatric patients, such as elderly hip fracture patients, and is associated with a poor prognosis. This study investigated (1) the prevalence of dysphagia based on clinical screening and a video-fluoroscopic swallowing study (VFSS), (2) the risk factors of dysphagia, and (3) the prognostic implications of dysphagia in elderly patients (≥ 65 years) undergoing hip fracture surgery.

**Methods:**

In this retrospective study, data from 393 female and 153 male patients ≥65 years of age who underwent surgery for a hip fracture between 2015 and 2018 were analysed. Patients who were considered at high risk of dysphagia after screening underwent a VFSS. To identify risk factors of dysphagia, demographic factors, the American Society of Anesthesiologists classification, past medical history, known risk factors of dysphagia, and factors associated with surgery were analysed using a binary logistic regression model. Odds ratios (ORs) of dysphagia for having poor prognosis including postoperative pneumonia, intensive care unit (ICU) admission, and death within 6 months after surgery were obtained by logistic regression. The association of postoperative pneumonia with poor prognosis was also analysed.

**Results:**

Dysphagia was seen in 5.3% of hip fracture patients. In multivariate regression analysis, a serum albumin level < 3.5 g/dL was identified as a risk factor for dysphagia (OR [95%CI] = 3.13 [1.40, 7.01]). Dysphagia was identified as a risk factor for postoperative pneumonia in regression analysis after adjustment (OR [95%CI] = 3.12 [1.05, 9.27]). Postoperative pneumonia was significantly associated with ICU admission (OR [95% CI] = 4.56 [1.85, 11.28]) and death within 6 months after surgery (OR [95% CI] = 2.56 [1.03, 6.33]).

**Conclusions:**

Dysphagia in elderly hip fracture surgery patients was associated with postoperative pneumonia, a risk factor for poor outcomes including ICU admission and death within 6 months after surgery. A serum albumin level < 3.5 g/dL was identified as a risk factor for dysphagia. Therefore, diagnostic testing should be performed to detect dysphagia, especially in patients with a low serum albumin level. Finally, particular care should be taken to prevent postoperative complications in patients with dysphagia.

## Background

Dysphagia, or disordered oropharyngeal swallowing, is a common finding in frail elderly patients caused by age-related physiological changes in swallowing, including a reduced neuronal coordination of the swallowing process, and decreased digestive tract motility due to decreases in muscle mass and connective tissue elasticity [1–3]. Dysphagia may lead to malnutrition, dehydration, and aspiration, which is the misdirection of oropharyngeal contents into the respiratory tract [3]. Aspiration in frail elderly patients can lead to aspiration pneumonia, a common and fatal postoperative complication. Therefore, a relationship of dysphagia with increases in mortality and length of hospital stay has been identified in hospitalized patients [4].

Osteoporotic hip fracture is a major health problem due to its association with a high mortality rate and its negative influence on the quality of life [5, 6]. Moreover, as populations age, osteoporotic hip fracture patients become more common [7]. Elderly patients with hip fractures are usually those who are frail and often present with an impaired functional status across several health metrics [8, 9]. In frail patients, an acute illness or procedure, including hip fracture surgery, may worsen an already reduced functional reserve and thus increase patient vulnerability to dysphagia [10]. A previous report identified a high prevalence of dysphagia in hip fracture patients [11]. A negative impact of aspiration pneumonia, which is closely related to dysphagia, on the prognosis of hip fracture patients has also been noted [12]. Therefore, the detection of dysphagia is an important factor in determining the prognosis of elderly patients with hip fracture.

Although a video-fluoroscopic swallowing study (VFSS) is considered the gold standard for diagnosing dysphagia, previous studies of dysphagia in hip fracture patients have relied on clinical methods to obtain a diagnosis [12, 13, 14]. Therefore, in the present study, we investigated (1) the prevalence of dysphagia using clinical screening and VFSS, (2) the risk factors for dysphagia, and (3) the prognostic implications of dysphagia in elderly (≥ 65 years) patients undergoing hip fracture surgery.

## Methods

This study was conducted with the approval of the Institutional Review Board. Informed consent was waived due to the retrospective design of the study. A total of 570 patients ≥65 years of age who underwent surgery for hip fracture (femoral neck fracture and intertrochanteric fracture) at the senior author’s institution (tertiary referral hospital) in Seoul, Republic of Korea between March 2015 and May 2018 were retrospectively analysed. Patients Cases of ipsilateral or contralateral hip fracture dislocation (1 patient), acetabular fracture or pelvic ring injury (3 patients), and femoral shaft fracture (1 patient) were not included in this study. Cases of pathologic fracture caused by neoplasms (11 patients) were also excluded, as were those presenting with pneumonia at the time of admission (7 patients). In addition, one patient whose medical records were insufficient for the analysis was not included. Therefore, 546 hip fracture patients (274 femoral neck fractures and 272 intertrochanteric fractures) constituted the study cohort.

### Diagnosis of dysphagia

Beginning in March 2015, the institution where the current study was performed implemented a protocol to detect oropharyngeal dysphagia and prevent postoperative pneumonia. Patients were screened for dysphagia at the time of admission to the ward. Thus, patients with a history of aspiration pneumonia or who complained of symptoms of aspiration, such as coughing or hoarseness during a meal, or with abnormalities (choking or difficulty drinking) in a water swallowing test using 30 mL of water at normal temperature [15], were considered at high risk of dysphagia and underwent a VFSS.

VFSS was performed by an experienced rehabilitation physician, assisted by a radiologic technician, in the fluoroscopic laboratory immediately after a patient assigned to the high-risk dysphagia group. Various boluses (3, 6, and 9 mL) were used in this study. Initially, a thick, curd-type yogurt was given to the patient. If there no dysphagia occurred, semisolid rice gruel was provided. The patient then received boiled rice in solid and liquid forms. Finally, 30 mL of diluted barium was provided. Patients were diagnosed as dysphagic if they showed any of the following abnormalities: 1) impaired mastication, lingual motion, or lip closure during the oral phase; 2) decreased pharyngeal contraction or swallowing reflex during the pharyngeal phase; 3) decreased laryngeal contraction, decreased laryngeal closure, or epiglottic inversion during the laryngeal phase; or 4) upper oesophageal sphincter dysfunction or decreased oesophageal contraction.

### Analysis of risk factors

The following demographic data were recorded: age, gender, body mass index (BMI), and indicators of the general condition of the patient, including the American Society of Anesthesiologists (ASA) classification. The medical histories of the patients, including stroke, dementia, and smoking, were assessed together with surgery-related factors (duration of the operation, interval between injury and surgery, method of anaesthesia, and surgical technique).

Results of blood chemistry tests, including albumin, blood urea nitrogen (BUN), and creatinine levels at admission, were reviewed. Blood was collected just after the fracture was confirmed in the emergency department. The serum albumin level was used as a marker of malnutrition, with a cut-off value of 3.5 g/dL. The BUN/creatinine ratio served as a marker for dehydration, with a cut-off value of 20:1.

### Prognostic implications of dysphagia

The prognostic implications of dysphagia were assessed based on a review of the patient’s medical records; specifically, the frequency of postoperative pneumonia, admission to the intensive care unit (ICU), death within 6 months after surgery. Postoperative pneumonia was diagnosed by radiologic study, including chest radiographs and computed tomography, and from the medical records, including consultations with a pulmonologist. Death within 6 months after surgery could be determined by reference to the end date of national health insurance for each patient.

### Statistical analysis

Categorical variables were compared using the chi-squared test. The chi-square test had 1 degree of freedom, since all categorical variables had two categories. Continuous variables were compared between groups using the Student’s t-test. The t-test had 544 degrees of freedom, since the number of patients included in the analysis was 546. The risk factors for dysphagia were analysed using a binary logistic regression model. Odds ratios (ORs) with 95% confidence intervals (CIs) were calculated.

A binary logistic regression analysis was performed to evaluate the association of dysphagia with postoperative pneumonia, ICU admission, and death within 6 months after surgery. The correlation of postoperative pneumonia, which is closely associated with dysphagia, with ICU admission and death within 6 months after surgery was also analysed.

A *P*-value < 0.05 was considered to indicate statistical significance. SPSS version 24.0 (IBM Corp., Armonk, NY) was used for statistical analyses.

## Results

Among the 546 patients, VFSS was performed in 40 patients (7.3%) who were considered to be at high risk for dysphagia, and dysphagia was diagnosed in 72.5% (29 of 40) of the patients. Thus, the prevalence of dysphagia among 546 elderly patients undergoing hip fracture surgery was 5.3% in our analysis.

Among the 29 patients with dysphagia, there were 14 males and 15 females with a mean age of 82.3 years (range: 72–95 years). Among patients without dysphagia, there were 139 males and 378 females with a mean age of 80.1 years (range: 64–104 years). The demographic data and risk factors of the two groups are summarized in Table 1.

**Table 1:** Demographic data and risk factors of dysphagia in hip fracture patients ≥65 years of age

Variable	Total	Dysphagia	Non-dysphagic			
	(*N* = 546)	(*n* = 29)	(*n* = 517)			
	Mean (SD) or *n* (%)	Mean (SD) or *n* (%)	Mean (SD) or *n* (%)	Chi-square	Effect size	*P*
Age (years)	80.3 (7.0)	82.3 (6.0)	80.1 (7.0)		0.307	0.109
Gender (male/female)	153/393	14/15	139/378	6.229	0.108	0.013
BMI (kg/m^2^)	22.1 (4.3)	20.6 (3.6)	22.2 (4.3)		0.370	0.053
ASA classification ≥3	295 (54.0%)	23 (79.3%)	272 (52.6%)	7.881	0.120	0.005
Stroke	90 (16.5%)	9 (31.0%)	81 (15.7%)	4.710	0.093	0.030
Dementia	86 (15.8%)	8 (27.6%)	78 (15.1%)	3.233	0.077	0.072
Delirium	38 (7.0%)	5 (17.2%)	33 (6.4%)	5.000	0.096	0.025
Smoking	28 (5.1%)	5 (17.2%)	23 (4.4%)	9.852	0.134	0.002
Fracture type				1.731	0.056	0.188
Femoral neck	274 (50.2%)	18 (62.1%)	256 (49.5%)			
Intertrochanteric	272 (49.8%)	11 (37.9%)	261 (50.5%)			
Operation time (min)	110.6 (24.1)	116.8 (39.4)	109.7 (22.9)		0.298	0.122
Interval between injury and operation ≥2 days	70 (12.8%)	7 (24.1%)	63 (12.2%)	3.510	0.080	0.061
Method of anaesthesia				0.680	0.035	0.410
General	242 (44.3%)	15 (51.7%)	227 (43.9%)			
Spinal	304 (55.7%)	14 (48.3%)	290 (56.1%)			
Surgical technique				2.976	0.074	0.084
Arthroplasty	254 (46.5%)	18 (62.1%)	236 (45.6%)			
Internal fixation	292 (53.5%)	11 (37.9%)	281 (54.4%)			
Malnutrition (Albumin< 3.5 g/dL)	110 (20.1%)	14 (48.3%)	96 (18.6%)	15.673	0.120	0.005
Dehydration (BUN/creatinine > 20)	355 (65.0%)	21 (72.4%)	334 (64.6%)	0.958	0.042	0.391

*Abbreviations*: *BMI* body mass index, *ASA* American Society of Anesthesiologists; BUN, blood urea nitrogen

*P =* Differences between patients with and without dysphagia

To analyse categorical variables, the Chi square test was performed and the phi coefficient was calculated for the effect size

To analyse continuous variables, te t-test was used and Cohen’s d was calculated for the effect size

Male gender, BMI, ASA classification ≥3, history of stroke, smoking, and serum albumin level < 3.5 g/dL were significant risk factors for dysphagia in the regression analysis without adjustment and were thus included in the multivariate regression model. Multivariate analysis showed that a serum albumin level < 3.5 g/dL was an independent risk factor for dysphagia (OR = 3.13; 95% CI = 1.40–7.01, *P* = 0.005) (Table 2).

**Table 2:** Multivariate analysis of the risk factors for dysphagia in hip fracture patients ≥65 years of age

Variable	Analytic model	Odds ratio	95% CI	*P*
Gender (male)	Model 1	2.54	1.19–5.39	0.015
	Model 2	1.43	0.61–3.34	0.414
BMI	Model 1	0.90	0.81–1.00	0.045
	Model 2	0.96	0.86–1.07	0.427
ASA classification ≥3	Model 1	3.45	1.38–8.62	0.008
	Model 2	2.19	0.83–5.76	0.113
Stroke	Model 1	2.42	1.07–5.51	0.035
	Model 2	1.81	0.75–4.39	0.188
Smoking	Model 1	4.69	1.63–13.45	0.004
	Model 2	2.91	0.86–9.80	0.085
Albumin < 3.5 g/dL	Model 1	4.20	1.96–9.00	< 0.001
	Model 2	3.13	1.40–7.01	0.005

*Abbreviations*: *CI* confidence interval, *BMI* body mass index, *ASA* American Society Anesthesiologists

1. Model 1 was not adjusted

2. Model 2 included all six variables listed in the table

The associations of dysphagia and postoperative pneumonia with poor outcomes are shown in Table 3. After adjusting for age and gender (model 1), dysphagia showed a significant correlation with postoperative pneumonia and ICU admission. However, after adding variables regarding general condition (model 2) and mental status (model 3) as covariates, dysphagia was significantly related only to postoperative pneumonia dysphagia. Postoperative pneumonia was significantly associated with ICU admission and death within 6 months after surgery in all models.

**Table 3:** Association of dysphagia and postoperative pneumonia with poor prognosis in hip fracture patients ≥65 years of age

Variables	Analytic model	Odds ratio	95% CI	*P*
Dysphagia				
Postoperative pneumonia	Model 1	4.52	1.64–12.48	0.004
	Model 2	3.19	1.10–9.21	0.032
	Model 3	3.12	1.05–9.27	0.041
ICU admission	Model 1	2.34	1.02–5.36	0.045
	Model 2	1.48	0.61–3.59	0.392
	Model 3	1.55	0.62–3.88	0.352
Death^a^	Model 1	2.00	0.79–5.03	0.141
	Model 2	1.23	0.46–3.27	0.683
	Model 3	1.20	0.44–3.23	0.724
Postoperative pneumonia				
ICU admission	Model 1	5.59	2.52–12.42	< 0.001
	Model 2	3.60	1.51–8.59	0.004
	Model 3	4.56	1.85–11.28	0.001
Death^a^	Model 1	3.73	1.60–8.70	0.002
	Model 2	2.50	1.02–6.16	0.046
	Model 3	2.56	1.03–6.33	0.042

*Abbreviations*: *CI* confidence interval, *BMI* body mass index, *ASA* American Society Anesthesiologists, *ICU* intensive care unit

Variables included in the model were based on univariate analysis

Notes: ^a^ death within 6 months after surgery

1. Model 1 was adjusted for age and gender

2. Model 2 was adjusted for age, gender, BMI, ASA classification ≥3, and albumin level < 3.5 g/dL

3. Model 3 was adjusted for age, gender, BMI, ASA classification ≥3, albumin level < 3.5 g/dL, dementia, delirium, and method of anaesthesia

## Discussion

In our study, dysphagia was diagnosed in 5.3% of 546 hip fracture surgery patients ≥65 years of age. A low serum albumin level was identified as an independent risk factor for dysphagia. Dysphagia was significantly associated with postoperative pneumonia, which is a risk factor for ICU admission and death within 6 months of surgery.

The prevalence of dysphagia has been studied in various patient populations using different diagnostic methods [4, 11, 16]. In the study of Altman et al. [4], dysphagia was diagnosed in only 0.73% of hospitalized patients over 75 years of age based on a retrospective analysis of data from the National Hospital Discharge Survey. Studies based on a clinical diagnosis of dysphagia reported higher prevalence rates. For example, Cichero et al. [16] reported a 25–30% prevalence among acutely hospitalized patients who underwent clinical screening for dysphagia. In their study of dysphagia in hip fracture patients, Love et al. [11] reported a prevalence rate of 34% among patients ≥65 years of age based on clinical diagnosis by a speech pathologist. In our study, 7.3% of hip fracture patients were considered to be at risk of dysphagia and thus underwent VFSS, which is considered the gold standard in the diagnosis of dysphagia [13]. Among those who were screened, 72.5% (5.3% of all hip fracture patients) were diagnosed with dysphagia. However, dysphagia may have been underdiagnosed, since not all hip fracture patients underwent a VFSS.

In the logistic regression analysis performed in this study, malnutrition, defined as a serum albumin level < 3.5 g/dL, was significantly associated with dysphagia. Malnutrition has been considered a sequela of dysphagia, since impaired swallowing can interfere with food intake [14]. On the other hand, malnutrition can also cause dysphagia via its negative effects on muscles and nerves [15]. Malnutrition has been suggested as one of the major factors promoting sarcopenia, as well as decreased muscle mass and strength [17]. In addition, changes in nerve function, including a reduction of nerve conduction velocity in patients with malnutrition, has also been noted [18]. An association between decreased systemic muscle mass and reduced swallowing function has been reported [19, 20], where a well-coordinated nervous system is essential to harmonize the various muscle actions involved in the swallowing process [21]. Accordingly, a circular relationship between poor nutritional status, neuromuscular dysfunction, and dysphagia was suggested [22]. Malnutrition is both a risk factor for, and a consequence of, dysphagia.

Our results demonstrate the negative implications of dysphagia with respect to the prognosis of hip fracture patients. Previous studies also reported poor prognosis in patients with dysphagia [4, 23]. In the above-cited study of Altman et al. [4], hospitalized patients with dysphagia had a higher rate of aspiration pneumonia, a longer hospitalization, and a higher proportion of death. Moreover, Cabre et al. [23] reported a higher proportion of death in pneumonia patients with dysphagia. Several factors are associated with a worse prognosis for hip fracture patients with dysphagia. First, dysphagia is closely associated with pneumonia and aspiration pneumonia [1]. In hip fracture patients, pneumonia is the most common postoperative complication [24], and is associated with poor outcomes, including longer hospital stay and higher mortality [12, 25]. In the current study, dysphagia was a risk factor for postoperative pneumonia, which was in turn found to be a risk factor for ICU admission and death within 6 months after surgery. Second, as confirmed in this study, dysphagia is closely associated with malnutrition [22], which, in hip fracture patients, is a risk factor for a poor prognosis [26, 27]. Drevet et al. [27] reported that hip fracture patients with protein-energy malnutrition had longer hospital stays. In their study of hip fracture patients, Chung et al. [26] showed that malnutrition, assessed by measuring the serum albumin level, was associated with postoperative complications, a longer hospital stay, and a higher mortality rate. The negative impact on prognosis of malnutrition may be due to its association with frailty and a reduced physiologic reserve, as also seen under conditions of surgical stress, for example [28, 29]. Third, and related to the second reason, dysphagia is not a distinct disorder of the oropharynx, but rather one of the phenotypes of frailty syndrome, which includes sarcopenia. Therefore, patients with dysphagia are likely to have comorbidities or other conditions affecting their prognosis. Singh et al. [30] demonstrated that, in patients who underwent spinal surgery, dysphagia was associated with age > 65 years, weight loss, and comorbidities including anaemia, neurological disorders, solid tumours, and fluid/electrical disorders. Maeda and Akagi [31] also showed that a low skeletal muscle mass and poor performance status were associated with dysphagia. In our study, patients with dysphagia had a higher ASA classification grade, and thus a larger number of comorbidities, as well as a poorer general condition than those without dysphagia.

There were several limitations to the current study. First, VFSS, a diagnostic study for oropharyngeal dysphagia, was not performed in all patients. Although all patients were screened following a VFSS, the screening method used may not have been able to detect patients with silent aspiration [32]. Therefore, the reported prevalence of dysphagia may have been lower than the actual prevalence, where this bias could have affected the results. Second, since our study was retrospective and included patients who were operated on at a referral hospital, selection bias may have affected the results. Third, the case-control study design used for identifying risk factors of dysphagia did not allow for the detection of causal relationships. Lastly, the serum albumin level may not represent the nutritional status of patients with inflammatory stress [33]. The Mini Nutritional Assessment would be preferable for assessing nutritional status. However, serum albumin level was used for this retrospective study since those data were available; moreover, it has been noted as a risk factor for poor outcomes in hip fracture patients [26, 34].

Despite these limitations, a strength of this study was that it assessed not only the prevalence but also the prognostic implications of dysphagia in elderly patients with hip fracture. In addition, the diagnosis was made using VFSS, the current gold standard for the diagnosis of dysphagia [13].

## Conclusion

Dysphagia in elderly hip fracture surgery patients was associated with postoperative pneumonia, a known risk factor for poor outcomes including ICU admission and death within 6 months after surgery. A serum albumin level < 3.5 g/dL was identified as an independent risk factor for dysphagia. Therefore, diagnostic testing should be performed to detect dysphagia, especially in patients with low serum albumin levels. Finally, particular care should be taken to prevent postoperative complications in patients with dysphagia.

## Data Availability

The datasets used and/or analysed during the current study are available from the corresponding author on reasonable request.

## Abbreviations

ASA: American Society of Anesthesiologists
BMI: Body mass index
BUN: Blood urea nitrogen
CI: Confidence intervals
ICU: Intensive care unit
OR: Odds ratio
VFSS: Video-fluoroscopic swallowing study
